# Minimal effective dose of ultrasound-guided rectus sheath block to reduce oral analgesic requirement after ambulatory laparoscopic tubal resection: a randomized controlled superiority trial

**DOI:** 10.1186/s13063-022-06158-3

**Published:** 2022-03-21

**Authors:** Sirikarn Siripruekpong, Jinsupha Aphinyankul, Thavat Chanchayanon, Maliwan Oofuvong, Jatupon Pakpirom, Chainarong Choksuchat, Patrapon Packawatchai, Jumras Na Klongdee

**Affiliations:** 1 Department of Anesthesiology, Faculty of Medicine, Prince of Songkla University, Songkhla, 90110 Thailand; 2 Department of Obstetrics and Gynecology, Faculty of Medicine, Prince of Songkla University, Songkhla, 90110 Thailand

**Keywords:** Rectus sheath block, Ambulatory surgery, Laparoscopic tubal resection, Oral analgesic requirement, Time to first oral analgesia, Pain score

## Abstract

**Background:**

The effective dose during ultrasound-guided rectus sheath block (URSB) for reducing pain after laparoscopic tubal ligation is reported to be 100 mg of 0.25% bupivacaine. We examined the minimal effective dose of 0.25% bupivacaine for URSB on oral analgesic requirement after ambulatory single-port laparoscopic tubal resection.

**Methods:**

A prospective, randomized controlled, superiority trial was conducted among patients who had been scheduled for ambulatory laparoscopic tubal resection between September 2015 and January 2019 at a tertiary care hospital in southern Thailand. Anesthesia was induced following protocol. The intervention group was allocated to receive a bilateral URSB using 10 ml of 0.25% bupivacaine on either side after intubation (total 50 mg) while the control group did not receive the sham block. Patients and assessors were blinded to the study intervention. All patients received a multimodal analgesia regimen as follows: fentanyl and ketorolac intraoperatively and fentanyl and oral acetaminophen at the post-anesthetic care unit. Postoperative oral analgesic requirement (acetaminophen and/or ibuprofen) at home was the primary outcome. Postoperative time to first analgesic requirement, oral analgesia (acetaminophen/ibuprofen), and pain score at 6 and 24 h were accessed via telephone interviews. Percentage, effect size (ES), and 95% confidence interval (CI) were presented.

**Results:**

A total of 66 out of 79 eligible patients were analyzed (32 intervention, 34 control). Intraoperative fentanyl consumption was significantly lower in the intervention group (*ES* [95% *CI*]: 0.58 [0.08, 1.07] mcg, *p* = 0.022). Time to first oral analgesia in the intervention group was significantly longer than that of the control group (*ES* [95% *CI*]: 0.66 [0.14, 1.16] h, *p* = 0.012). The proportion of oral analgesia requirement at 24 h after surgery in the control group was significantly higher than that in the intervention group (97% vs 75%, *p* = 0.012). Pain scores at 6 and 24 h were similar in both groups although slightly lower in the intervention group (*ES* [95% *CI*]: 0.22 [−0.26, 0.71], *p* = 0.368 and 0.33 [−0.16, 0.81], *p* = 0.184, respectively).

**Conclusion:**

A dose of 0.25% bupivacaine 50 mg for URSB reduced the oral analgesic requirement at 24 h and prolonged the time to first analgesic requirement after ambulatory laparoscopic tubal resection.

**Trial registration:**

Thaiclinicaltrials.orgTCTR20150921002. Registered on 18 September 2015

## Background

Balanced analgesic techniques in ambulatory laparoscopic tubal ligation have been reported to have better postoperative pain control and increased speed of recovery [1]. Development of pain after laparoscopic sterilization is associated with the rapid distension of the peritoneum from inflammatory mediators due to traumatic traction of the nerves and blood vessels. It is also associated with occlusion of the fallopian tube on autonomic innervation via the mesosalpinx [2–4] and shoulder tip pain from the phrenic nerve excitation from carbon dioxide insufflation [5, 6]. A rectus sheath block (RSB) can provide analgesia around the midline including the surgical incisions near the umbilicus [7, 8]. The effective dose to reduce pain after ambulatory single-port laparoscopic tubal ligation under RSB was reported to be 0.25% bupivacaine 100 mg (40 ml) [9]. A lower dose of 0.25% bupivacaine to perform RSB under ultrasound-guided may provide effective postoperative analgesia and reduce the risk of side effects due to the higher dose of bupivacaine. Therefore, we examined the minimal effective dose of 0.25% bupivacaine 50 mg (20 ml) for ultrasound-guided rectus sheath block (URSB) on oral analgesic requirement and pain after ambulatory single-port laparoscopic tubal resection (LTR) combined with multimodal analgesia.

## Materials and methods

A randomized controlled, double-blinded, parallel superiority trial was conducted after approval from the Ethics Committee of the Faculty of Medicine, Prince of Songkla University, Thailand (EC 58155081). All patients gave their written informed consent after receiving essential information of the study objectives. Patients were invited to participate between September 2015 and January 2019 at the operating theater of Songklanagarind Hospital. The Thaiclinicaltrials.org (TCTR20150921002) was submitted and released on 18 September 2015 and 21 September 2015, respectively, while the first patient enrollment was on 18 September 2015. We enrolled American Society of Anesthesiologists (ASA) physical status I–II patients who were scheduled for ambulatory LTR. Those with a history of drug allergy (propofol, cisatracurium, fentanyl, bupivacaine, ondansetron, ketorolac, neostigmine, atropine, acetaminophen), a previous history of chronic pain, and a history of gastroesophageal reflux disease were excluded. The DOI link by Protocols.io is dx.doi.org/10.17504/protocols.io.bxq9pmz6.

### Study protocol

Patients were randomly assigned into two groups: a control group and an intervention group, which was achieved using a computer-generated, randomization table by block of 4 with an allocation ratio of 1:1 between the groups. Treatment assignment by a research assistant (JNK) was sealed in opaque envelopes for the participants who showed up in the operating theater on the day of surgery. Participants were enrolled by a nurse investigator (PP) at the perioperative clinic at least 1 month before surgery. On the day of surgery, the patient’s data was recorded by anesthetist nurses. After, standard monitoring (electrocardiogram, non-invasive arterial blood pressure, pulse oximeter, and end-tidal CO_2_ concentration) was applied. Hemodynamic parameters such as blood pressure and heart rate were monitored every 5 min. The electrocardiogram, pulse oximeter, and end-tidal CO_2_ concentration were real-time continuously monitored. General anesthesia was induced by propofol (2–3 mg/kg), fentanyl (2 mcg/kg), and cisatracurium (0.15 mg/kg) intravenously. When the intubation was completed, intravenous ketorolac was given. Patients were blinded to the treatment allocation since it was given after the patients were anesthetized before commencing the operation. The intervention group received bilateral URSB whereas the control group received the same preparation with an ultrasound scan similar to the intervention group except for the needle injection part. A sham block was not performed since our local Ethics Committee expressed concerns about the invasive procedure with no added benefit provided to the subjects. Both groups received the multimodal analgesia regimen after the treatment intervention. The anesthetist nurse and the anesthesiologist in charge were asked to leave the operating theater during the intervention. Only the anesthesiologist who performed an ultrasound (SS or JP) and a nurse investigator (PP) remained in the theater during the intervention.

During the maintenance phase, sevoflurane, fentanyl, and cisatracurium were adjusted to keep the patient anesthetized at the discretion of the anesthesiologist in charge who was not aware of the allocated intervention. The operation was performed with a single-port 10-mm trocar transducer. The insertion of pneumoperitoneum with carbon dioxide was insufflated and the intraabdominal pressure was kept at less than 15 cmH_2_O. Thirty minutes before the end of the operation, ondansetron 4 mg was given intravenously to all patients. When the operation was completed, neostigmine 0.05 mg/kg and atropine 0.02 mg/kg were given for reversal of neuromuscular blockade as indicated.

The post-anesthetic care unit (PACU) nurses were not aware of the treatment intervention. Within the PACU, besides oral acetaminophen, postoperative pain scores, as well as any adverse events such as dizziness, nausea, or vomiting, were evaluated by an investigator (JA), who was not involved during the intraoperative period. The decision to discharge the patient home or transfer the patient to a ward was jointly made by a PACU anesthesiologist and the surgeon. After discharge home, an evaluation was performed by the same investigator (JA), who was not aware of the treatment allocation, via a telephone interview to assess oral analgesic requirement, pain score, and side effects at 6 and 6–24 h postoperatively.

### Treatment allocation protocol (Fig. 1)

**Fig. 1:**
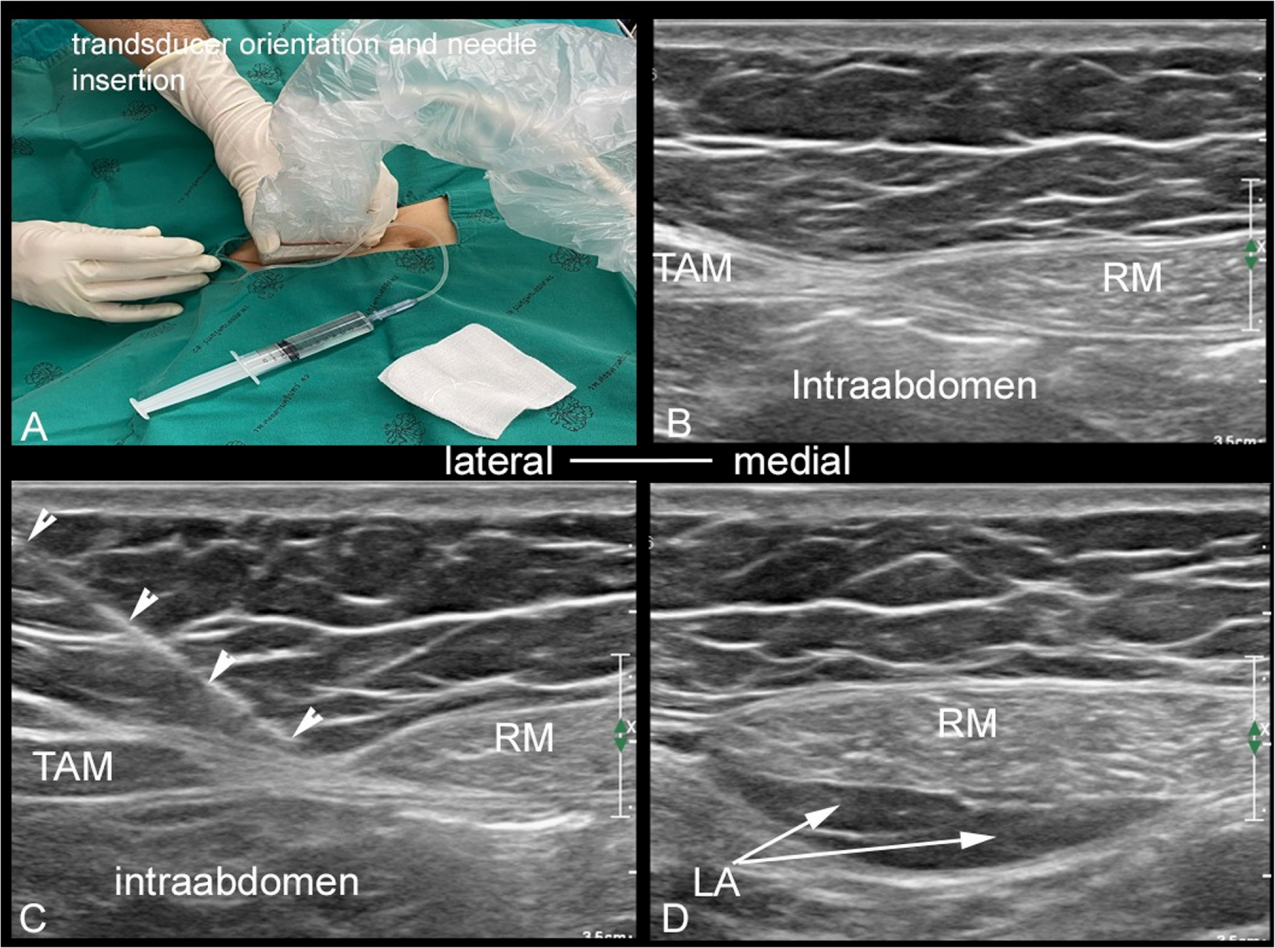
Ultrasound-guided rectus sheath block technique. *RM* rectus sheath muscle, *TAM* transversus abdominis muscle

After general anesthesia was established, a bilateral URSB (intervention group) or ultrasound scan (control group) was performed by two regional anesthesiologists (SS, JP) with at least 3 years of experience in ultrasound-guided peripheral nerve block. The procedure was performed using an aseptic technique, and a linear ultrasound transducer (5–12 MHz) was prepared with a sterile drape. The transducer was placed at the mid-abdomen in transverse scanning just above the umbilicus. It was then moved to either the left or right side to identify the lateral border of the rectus muscle connecting with the transversus abdominis muscle (Fig. 1A, B). Among the intervention group, the block needle (SonoTAP 22Gx80mm; Pajunk, Germany) was then inserted in-plane approach from the lateral to the medial direction targeting the needle tip between the rectus muscle and the transversalis fascia (Fig. 1C). Normal saline was used to accomplish hydrolocation for confirming the correct facial plane and then 0.25% bupivacaine 10 ml was incrementally injected (Fig. 1D). The other side was repeated using the same procedure and another 10 ml of 0.25% bupivacaine was used for the URSB.

### The multimodal analgesia protocols

The multimodal analgesia regimen consisted of 3 phases: the intraoperative period, during recovery at PACU, and either at home or the ward. During the intraoperative period, patients received intravenous fentanyl 2 mcg/kg before intubation and intravenous ketorolac 0.5 mg/kg before the assigned treatment was given. Additional fentanyl 0.5 mcg/kg was given intravenously depending on the discretion of the anesthesiologist in charge. All patients received oral acetaminophen 15–20 mg/kg after arrival at the PACU. Intravenous fentanyl 0.5 mcg/kg was given every 15 min if the maximum pain score exceeded 3. After discharge from the PACU, patients could take acetaminophen 500 mg orally (for BW < 50 kg) or 1000 mg orally (for BW > 50 kg) every 6 h if their maximum pain score exceeded 3. One hour after acetaminophen was taken (7 h postoperatively), if the patient’s pain score still exceeded 3, ibuprofen 400 mg orally could be taken for pain relief and every 8 h.

### Outcomes of interest

The primary outcome was the proportion of patients who received oral analgesic at home or the ward 1–24 h after surgery. The secondary outcome was the time to first oral analgesic requirement (hours) at home or the ward. The tertiary outcome was the maximum pain score by verbal numerical rating scale (VNRS) at 0, 1, 6, and 24 h after surgery.

### Sample size determination

For the primary objective, the sample size was estimated based on a 30% reduction in the proportion of oral analgesic requirement between the control (0.97) and the intervention groups (0.67) under a level of significance of 0.05 and 80% power to detect this difference. A total of 35 patients per group were required under a 10% dropout rate. For the secondary objective, the sample size was estimated based on a study by Gurnaney et al. [10]. The difference in time to first oral analgesic requirement between the control (25 min) and the intervention (50 min) groups was 25 min with a standard deviation of 37 min under a level of significance of 0.05 and 80% power to detect this difference. The required sample size, which included a 10% dropout rate, was 78 patients. Therefore, the final enrollment would be at least 78 patients to accomplish both objectives.

### Statistical analysis

The analysis followed the intention-to-treat principle. Statistical analysis was performed using R (version 4.0.2, R Core Team, Vienna). Continuous variables were presented as mean and standard deviation (SD) for normally distributed variables and median and interquartile range (IQR) for non-normally distributed variables. Normally distributed continuous variables were analyzed via a *t*-test, while non-normally distributed continuous variables were analyzed using Wilcoxon’s rank-sum test. Effect size by Cohen’s statistic was also performed. Categorical variables were presented as frequency with percentage. For analyzing the difference in outcomes between categorical variables, a chi-square test or Fisher’s exact test was used as appropriate. Time to first oral analgesic-free survival between the control and intervention groups was compared using the log-rank test. Changes in pain score at 0, 1, 6, and 24 h were compared using the generalized estimating equations method. Odds ratio (OR), effect size (ES), and 95% confidence interval (CI) were presented for the main outcomes. A *p*-value less than 0.05 was considered statistically significant.

## Results

**Fig. 2:**
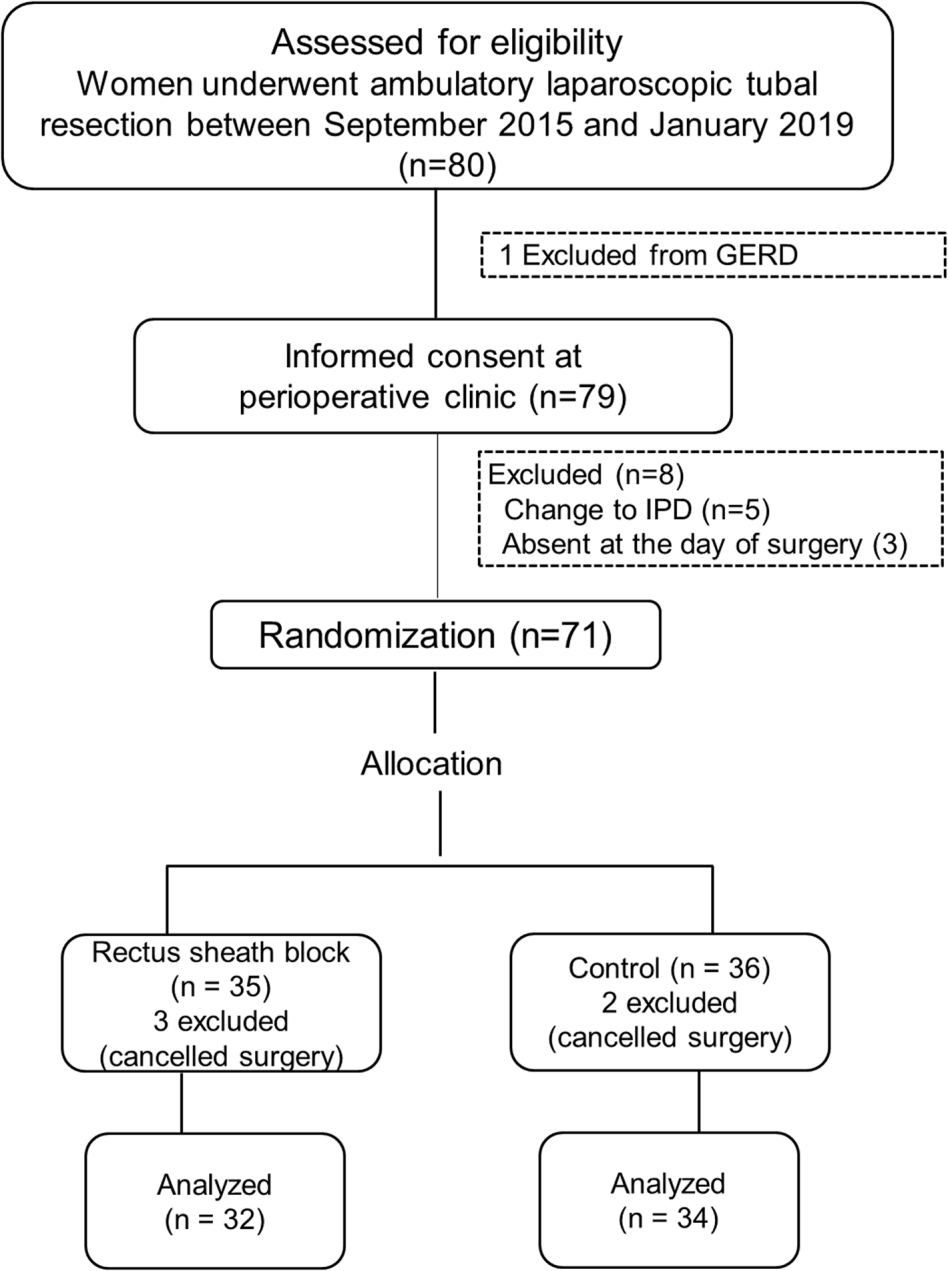
Consort flow of the study. *GERD* gastroesophageal disease, *IPD* inpatient department

Seventy-eight patients were enrolled in the study from September 2015 to December 2018, of which 34 patients in the control group and 32 in the intervention group were analyzed (Fig. 2). A comparison of the demographic data between the two groups is shown in Table 1. There were no statistically significant differences between the two groups in terms of age, weight, body mass index, ASA physical status, and underlying diseases. Additionally, no subject was currently using any medication.

**Table 1:** Patient demographic data

Demographic data	Control (***n*** = 34)	URSB (***n*** = 32)	***p***-value
Age (years), mean (SD)	36.5 (4.3)	36.6 (3.3)	0.948
Body weight (kg), mean (SD)	59.6 (12)	59.9 (8.9)	0.922
Height (cm), mean (SD)	157.8 (6.1)	159 (4.9)	0.380
Body mass index (kg/m^2^), mean (SD)	23.8 (4)	23.7 (3.3)	0.876
ASA classification			0.475
I	13 (38.2)	16 (50.0)	
II	21 (61.8)	16 (50.0)	
Underlying disease			
Respiratory system (recent URI, AR)	3 (8.8)	2 (6.2)	1
Cardiovascular system (abnormal ECG)	3 (8.8)	0 (0)	0.239
Endocrine system (obesity, thyroid disease)	4 (11.8)	2 (6.2)	0.673
Other systems (anemia, dyslipidemia)	15 (44.1)	11 (34.4)	0.577

Data was presented as frequency (%) unless stated otherwise

*Abbreviations*: *AR* allergic rhinitis, *ASA* American Society of Anesthesiologists, *ECG* electrocardiography, *URSB* ultrasound-guided rectus sheath block, *SD* standard deviation, *URI* upper respiratory tract infection

Table 2 compares anesthesia information at the intraoperative period and at the PACU between the two groups. During the intraoperative period, operation time, intraabdominal pressure during pneumoperitoneum, and intravenous ketorolac consumption were not significantly different between the two groups. However, the average fentanyl consumption in the control group was significantly higher than that of the intervention group (*ES* [95% *CI*]: 0.58 [0.08, 1.07] mcg, *p* = 0.022). There was no difference in the incidence of bradycardia and hypotension between the two groups (*p* = 0.705).

**Table 2:** Anesthesia information at intraoperative period and at the post-anesthetic care unit

Anesthesia information	Control (***n*** = 34)	URSB (***n*** = 32)	***p***-value
Duration of operation (minutes), median (IQR)	36.5 (25, 45)	32.5 (25, 45)	0.982
Intraabdominal pressure during pneumoperitoneum (cmH_2_O), median (IQR)	15 (14.2, 15.0)	15 (14.0,15.0)	0.405
**Total anesthetic agent consumption**			
Intraoperative period			
Total fentanyl (mcg), mean (SD)	129.4 (35.6)	111.7 (24.6)	0.022*
Fentanyl (mcg)/kg, mean (SD)	2.2 (0.6)	1.9 (0.5)	0.022*
Ketorolac (mg), median (IQR)	30 (30, 30)	30 (30, 30)	0.938
Post-anesthetic care unit			
Fentanyl	30 (88.2)	26 (81.2)	0.505
Total fentanyl (mcg), median (IQR)	87.5 (52.5, 123.8)	75 (25, 131.2)	0.796
Fentanyl (mcg)/kg, median (IQR)	1.5 (0.8, 2)	1.3 (0.5, 2.4)	0.748
Acetaminophen	34 (100)	32 (100)	1
Intraoperative complications	3 (8.8)	4 (12.5)	0.705
Bradycardia	1 (2.9)	4 (12.5)	0.190
Hypotension	2 (5.9)	2 (6.2)	1
Post-anesthetic care unit complications	10 (29.4)	7 (21.9)	0.676
Nausea/vomiting	6 (17.6)	3 (9.4)	0.477
Dizziness	5 (14.7)	5 (15.6)	1
Time to meet discharge criteria (minutes), mean (SD)	217.1 (64.1)	207.3 (74.7)	0.572
Destination upon discharge from PACU, *n* (%)			0.705
Home	31 (91.2)	28 (87.5)	
Ward	3 (8.8)	4 (12.5)	
Reason for admission, *n* (%)			
Pain	2 (5.9)	2 (6.3)	1
Dizziness	1 (2.9)	2 (6.3)	0.608

Data was presented as frequency (%) unless stated otherwise

*Abbreviations*: *IQR* interquartile range, *RSB* rectus sheath block, *SD* standard deviation, *PACU* postoperative care unit

*Unpaired *t*-test

While in the PACU, the total consumption of fentanyl and acetaminophen was not significantly different between the two groups. Additionally, there was no difference in average time to meet the discharge criteria within the PACU period (mean: 217 vs 207 min, *p* = 0.572). Seven patients were admitted to the hospital (3 in the control group and 4 in the intervention group), all due to pain and dizziness.

**Fig. 3:**
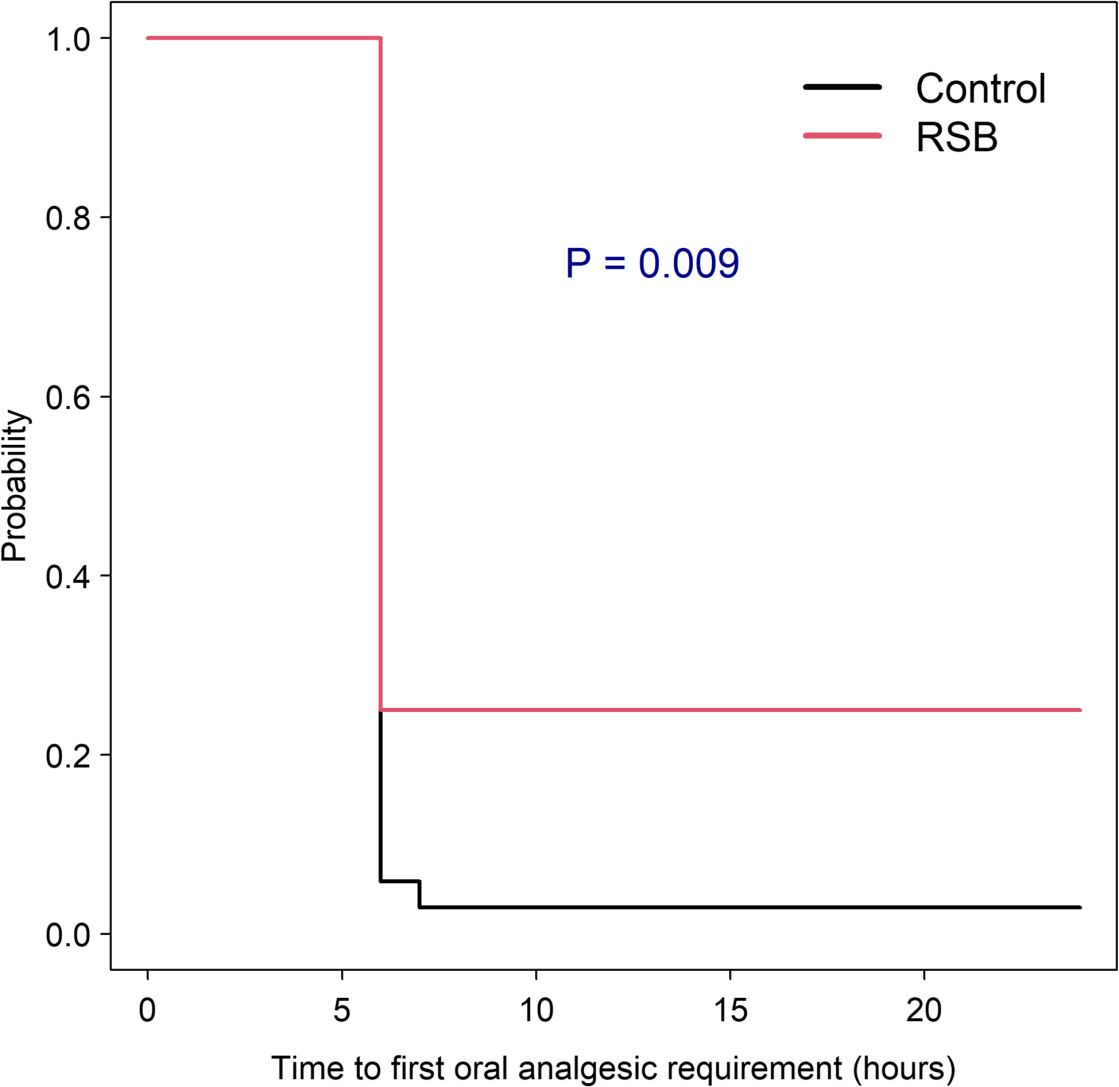
Kaplan–Meier curves of time to first oral analgesic requirement between the two groups. *RSB* rectus sheath block

At home/ward, the average time to first oral analgesia in the intervention group was significantly longer than that of the control group (*ES* [95% *CI*]: 0.66 [0.14, 1.16] h, *p* = 0.012). Figure 3 shows the Kaplan–Meier curves for time to first oral analgesic requirement among the two groups. The overall probability of being free of oral analgesic requirement at 24 h in the intervention group was significantly higher than that of the control group (0.25 [95% *CI* 0.137, 0.456] vs 0.030 [0.004, 0.203]).

**Fig. 4:**
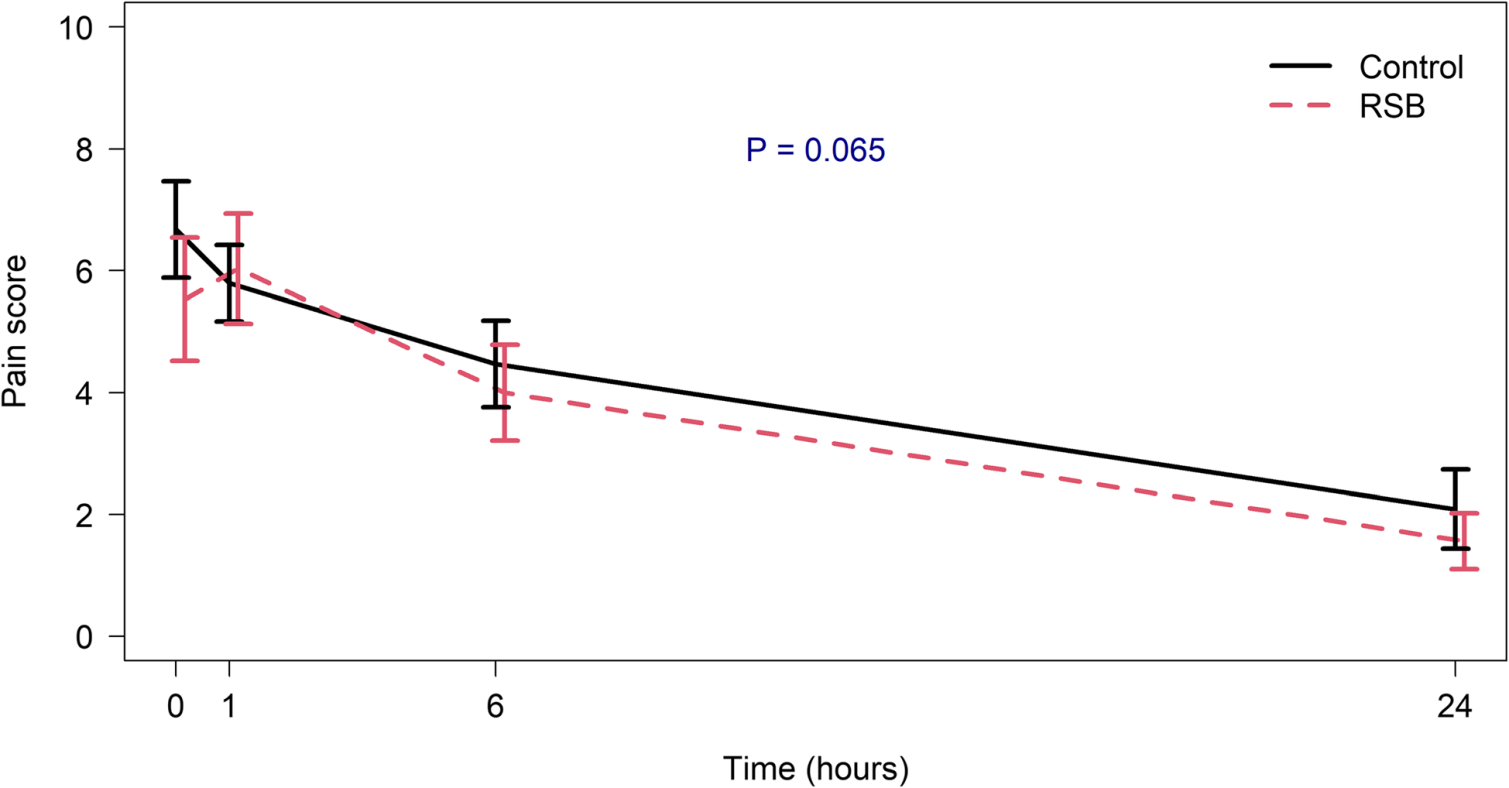
Comparison of postoperative pain scores at 0, 1, 6, and 24 h after surgery between the two groups. *RSB* rectus sheath block

Table 3 compares oral analgesic requirement, pain assessment, and side effects between the two groups at 6 and 24 h postoperatively. The proportion of oral analgesia requirement at 6 and 24 h after surgery in the control group was significantly higher than that of the intervention group (*OR* [95% *CI*]: 0.192 [0.018, 1.083], *p* = 0.041 and 0.094 [0.002, 0.777], *p* = 0.012, respectively). The total acetaminophen and ibuprofen consumption were not different at 1–24 h between the two groups (*p* = 0.486 and *p* = 0.938, respectively). The average pain scores at 6 and 24 h were slightly lower in the intervention group (*p* = 0.368 and 0.184, respectively). There was no significant difference in the overall postoperative pain score between the two groups (*p* = 0.065) (Fig. 4). The prevalence of nausea and vomiting at home/ward showed no statistically significant difference between the two groups. Seven patients who were admitted because of moderate pain/dizziness after surgery were relieved by supportive treatment, oral fluids, and oral analgesic control. All patients were discharged the next day.

**Table 3:** Comparison of oral analgesic requirements and pain assessment between the two groups

Outcome, ***n*** (%)	Control (***n*** = 34)	URSB (***n*** = 32)	***OR*** (95% ***CI***)	***p***-value
Oral analgesic requirement at 6 h	32 (94.1)	24 (75.0)	0.192 (0.018, 1.083)	0.041*
Oral analgesic requirement at 24 h	33 (97.1)	24 (75)	0.094 (0.002, 0.777)	0.012*
Nausea/vomiting at 6 h	4 (11.8)	5 (15.6)	1.38 (0.27, 7.72)	0.730
Nausea/vomiting at 24 h	2 (5.9)	4 (12.5)	2.26 (0.30, 26.75)	0.420
**Other outcomes, mean (SD)**	**Control (*n*** **= 34)**	**URSB (*n*** **= 32)**	**Effect size (95%** ***CI*)**	***p*-value**
Time to first oral analgesia at home (h)	6.6 (3.1)	10.5 (7.9)	−0.66 (−1.16, −0.14)	0.012**
Acetaminophen at 6 h (mg)^†^	455.9 (396.3)	437.5 (470.9)	0.04 (−0.44, 0.53)	0.865
Acetaminophen at 24 h (mg)^†^	838.2 (967.0)	687.5 (748.7)	0.17 (−0.31, 0.66)	0.480
Ibuprofen at 6 h (mg)^†^	141.2 (217.6)	137.5 (193.0)	0.02 (−0.46, 0.50)	0.942
Ibuprofen at 24 h (mg)^†^	282.4 (423.9)	275.0 (343.6)	0.02 (−0.46, 0.50)	0.938
Postoperative VNRS pain score				
Immediate pain score (time 0)	6.7 (2.3)	5.5 (2.8)	0.45 (−0.04, 0.94)	0.072
1 h	5.8 (1.8)	6.0 (2.5)	−0.11 (−0.59, 0.38)	0.659
6 h	4.5 (2.0)	4.0 (2.2)	0.22 (−0.26, 0.71)	0.368
24 h	2.1 (1.9)	1.6 (1.3)	0.33 (−0.16, 0.81)	0.184

^†^Presented as median (SD) since median (IQR) were the same between the two groups

*Abbreviations*: *OR* odds ratio, *RSB* rectus sheath block, *SD* standard deviation, *VNRS* verbal numerical rating scale

*Fisher’s exact test, **unpaired *t*-test

## Discussion

We hypothesized that a minimal dose (50 mg) of 0.25% bupivacaine for bilateral URSB could reduce the postoperative oral analgesic requirement and prolong the time to first oral analgesia at home or the ward after receiving a laparoscopic tubal resection. We found that the intervention could significantly reduce the oral analgesic requirement at 24 h from 97 to 75% (*OR* [95% *CI*]: 0.09 [0.002, 0.777]) and prevented the need for oral analgesic for 10.5 h after the last dose of 15–20 mg/kg of oral acetaminophen. A lower dose of intraoperative fentanyl consumption in the URSB group (*p* = 0.022) could have arisen from the reduced pain and fewer hemodynamic changes during surgery from effective rectus sheath block.

Our results are consistent with a study by Shah among patients receiving laparoscopic tubal ligation where tramadol requirements in the first 12 h postoperatively were lower in those who received ultrasound-guided single injection RSB [9]. However, unlike in our study, URSB with 0.25% bupivacaine 100 mg (40 ml) could significantly reduce postoperative verbal analogue pain scores. The pain that patients mentioned in our study consisted of surgical site pain (somatic pain) and pelvic pain (visceral pain). We believe that tubal resection in our study caused more visceral pain than tubal ligation in Shah’s study, where deep visceral pain may not have been entirely covered by URSB since this type of block provides more somatic pain relief. In our study, the average immediate pain scores (time 0) after the operation was moderate in the intervention and control groups (5.5 and 6.7, respectively). At 6 and 24 h postoperatively, pain scores were lower in both groups since multimodal analgesia (intraoperative ketorolac and oral acetaminophen at PACU) were started for all patients. Since URSB can provide somatic pain relief for abdominal wall structures near the umbilicus superficial to the peritoneum via blockage of terminal branches of the 9th, 10th, and 11th intercostal nerves [7], trocar site pain (somatic pain) was alleviated by URSB. Therefore, 10 ml (25 mg) either side of 0.25% bupivacaine for URSB (somatic pain control) combined with multimodal analgesia (visceral pain control) could provide effective oral analgesic requirement up to 10.5 h compared to the control group (6.6 h) and decrease the risk of oral analgesia requirement at 24 h by a factor of 10 compared to those in the control group. Since all patients received oral acetaminophen at the PACU (phase 2, time 0 h) and intravenous ketorolac after intubation (phase 1, time −1 h), the Kaplan–Meier plot showed little variability among both groups since time to first oral analgesia in patients who required rescue dose occurred mostly at 6 h after surgery. However, the total acetaminophen consumption was not different at 24 h between the two groups, which might be because patients received different doses of acetaminophen individually depending on their weight and combination with ibuprofen may be inaccurate to compare analgesic equivalence between the two groups.

Previous studies reported that the main role of the URSB for midline abdominal surgery is to reduce postoperative pain and consumption of perioperative opioids [11–15]. To date, bilateral URSB has also been performed in other laparoscopic procedures such as cholecystectomy [16, 17] and gynecologic surgery [18, 19]. However, one case report [20] and the study by Shah [9] are the only reports of URSB in laparoscopic tubal ligation. Hariharan et al. [20] reported a bilateral URSB for a single-incision laparoscopic tubal ligation without general anesthesia in a cardiac patient. A recent meta-analysis (2021) reported that URSB could improve pain control for up to 12 h postoperatively and reduce opioid consumption without major adverse events among adults receiving laparoscopic surgery in both the inpatient and outpatient settings [21]. The effective dose of URSB varied from 75 to 100 mg of bupivacaine (0.5% 20 ml and 0.25% 40 ml) [9, 22] and ropivacaine (0.25% 30 ml and 0.5% 20 ml) [17–19], which were much higher than in our study. We did not use ropivacaine due to its unavailability.

In terms of time to meet the discharge criteria at the PACU, there was no statistically significant difference between the intervention and the control groups in our study (*p* = 0.57) due to there being no differences in postoperative pain and dizziness scores between both groups. A study by Hamill [23] found that patients in their intervention group spent less time in the PACU after inpatient laparoscopic appendicectomy (mean [SD]: 25 [15] vs 32 [17] min, *p* = 0.022). This may be because that study was an inpatient setting and ours was an outpatient setting where patients need to meet a phase 2 PACU discharge criteria (post-anesthetic discharge scoring system). Moreover, studies done in ambulatory surgery, both in adult and pediatric settings, did not report the total PACU time after laparoscopic tubal ligation [9] and umbilical hernia surgery [24, 25].

### Application of URSB for laparoscopic tubal resection

What we learnt from this study was that a lower dose of 0.25% bupivacaine (50 mg) for URSB provided good postoperative analgesia for up to 24 h by reducing oral analgesic requirement by 10 times compared to the control group. The average pain score in the intervention group 6 and 24 h postoperatively was lower by 0.5 points compared to the control group. Since URSB provided somatic pain relief at the trocar site while tubal resection caused moderate visceral pain in our study, early commencement of preemptive analgesia/multimodal analgesia (preoperative oral acetaminophen at the preoperative area in an outpatient setting or intravenous paracetamol) combined with a lower dose of 0.25% bupivacaine before starting the operation may provide effective postoperative pain control after laparoscopic surgery. If there are no experienced anesthesiologists to perform this block, we recommend the use of a multimodal analgesic regimen including local anesthetic infiltration at the trocar site to reduce postoperative pain and adverse events from opioids and other analgesics.

### Strengths and limitations

A strength of this study is that it was a randomized controlled, double-blind superiority trial where both the patient and assessor were blinded to the treatment allocation. To reduce the possibility of performance bias, we used two investigators (SS, JP) who had more than 3 years of experience using peripheral nerve blocks. Both investigators were unaware of the main outcomes (oral analgesia requirements). Despite this strength, our study has a few limitations. First, we did not use the sham block since it may be controversial and unethical and could lead to harm [26]. Second, we did not determine postoperative pain score at 12 h since a lower pain score might appear in the intervention group due to the longer effect of URSB. Lastly, since the subjects were quite healthy (ASA I–II) and we confined our study subjects to Asians, the results of this study may not be generalizable to less-healthy individuals or those from regions outside Asia.

## Conclusion

A minimal effective dose (50 mg) of 0.25% bupivacaine for ultrasound-guided rectal sheath block reduced the oral analgesic requirement at 6 and 24 h and prolonged the time to first analgesic requirement after laparoscopic tubal resection.

## Data Availability

The datasets used and/or analyzed during the current study are available from the corresponding author on reasonable request.

## Abbreviations

ASA: American Society of Anesthesiologists
CI: Confidence interval
ES: Effect size
IQR: Interquartile range
LTR: Laparoscopic tubal resection
OR: Odds ratio
PACU: Post-anesthetic care unit
RSB: Rectus sheath block
SD: Standard deviation
URSB: Ultrasound-guided rectus sheath block
VNRS: Verbal numerical rating scale
